# Prevalence of Moraxella Species Among Lower Respiratory Tract Infections in and Around Kanchipuram

**DOI:** 10.7759/cureus.107646

**Published:** 2026-04-24

**Authors:** Maheswari S, Sivasankari S, Senthamarai S, Akila K, Subha V J, Anitha S, Ambuja Sekhar

**Affiliations:** 1 Department of Microbiology, Meenakshi Medical College Hospital and Research Institute, Meenakshi Academy of Higher Education and Research, Kanchipuram, IND

**Keywords:** antimicrobial susceptibility, chronic obstructive pulmonary disease, lower respiratory tract infection, moraxella catarrhalis, respiratory isolates

## Abstract

Objective: This study aims to determine the proportion of *Moraxella catarrhalis* among bacterial isolates recovered from lower respiratory tract specimens and to assess the associated demographic and clinical characteristics, antimicrobial susceptibility pattern, and phenotypic clavulanate synergy positivity.

Methods: This prospective observational study was conducted in the Department of Microbiology at Meenakshi Medical College Hospital and Research Institute, Kanchipuram, Tamil Nadu, India, from December 2024 to December 2025. A total of 100 eligible respiratory specimens, including sputum, bronchoalveolar lavage, and bronchial washing samples, were analyzed. *M. catarrhalis* was identified by conventional phenotypic methods, including the hockey puck test. Antimicrobial susceptibility testing was performed using routine phenotypic methods, and susceptibility results were summarized descriptively. Data were analyzed using IBM SPSS Statistics for Windows, Version 29 (Released 2023; IBM Corp., Armonk, New York), and p < 0.05 was considered significant.

Results: Among 100 bacterial isolates recovered from eligible lower respiratory tract specimens, 16 isolates (16.0%) were identified as* M. catarrhalis*. Overall, isolate distribution differed significantly across organism categories χ² = 17.04, p = 0.009. Among *M. catarrhalis* isolates, 56.3% occurred in males, and the highest frequency was observed in patients older than 60 years, 37.5%. Chronic obstructive pulmonary disease was the most common associated clinical condition (31.3%), and diabetes mellitus was the most frequent risk factor (43.8%). Ampicillin showed the highest resistance (43.8%), followed by ciprofloxacin (31.3%) and cefotaxime (18.8%). All isolates were susceptible to azithromycin, and 93.8% were susceptible to amoxicillin-clavulanate. One isolate (6.3%) showed clavulanate synergy positivity.

Conclusion: *M. catarrhalis* constituted a clinically relevant proportion of bacterial isolates recovered from lower respiratory tract specimens in this setting. Its isolation was more frequent in older adults and in patients with chronic airway disease and diabetes mellitus. The observed resistance to ampicillin, along with notable resistance to ciprofloxacin and cefotaxime, supports careful laboratory identification and locally guided antimicrobial therapy.

## Introduction

Lower respiratory tract infections remain a major cause of morbidity across hospital and community settings, particularly among older adults and patients with chronic pulmonary disease [1]. Although classical pathogens such as *Streptococcus pneumoniae*, *Haemophilus influenzae*,* Klebsiella pneumoniae*, and non-fermenting gram-negative bacilli dominate routine diagnostic consideration, *Moraxella catarrhalis* has emerged from its earlier reputation as a harmless upper-airway commensal to an established respiratory pathogen with clear clinical relevance in adults [1,2].

Over the last several decades, microbiological and clinical evidence has shown that *M. catarrhalis* is capable of causing genuine lower respiratory tract infections, especially in individuals with chronic obstructive pulmonary disease (COPD), bronchiectasis, post-tubercular structural lung damage, advanced age, and other states of impaired airway defense [3,4]. This shift in understanding is important because organisms once dismissed as colonizers may, in fact, contribute to exacerbations, persistent inflammation, recurrent hospitalization, and avoidable antimicrobial misuse when not recognized accurately. In respiratory microbiology, therefore, the problem is not simply whether *M. catarrhalis* is isolated, but in whom it is isolated, under what clinical circumstances, and with what antimicrobial profile.

The pathogenic relevance of *M. catarrhalis* is especially compelling in chronic airway disease. Reviews of COPD microbiology have placed *M. catarrhalis* among the important bacterial organisms recovered during exacerbations, alongside *H. influenzae* and *S. pneumoniae*, while also emphasizing that persistent airway colonization may not be biologically inert because it can sustain maladaptive inflammation and contribute to disease progression [5]. More recent clinical work has reinforced this concern. Yagyu *et al.* (2025) [6] reported that prior lower-airway infection with *M. catarrhalis* was strongly associated with recurrent hospitalization for acute exacerbation of COPD, supporting the view that the organism may act as a marker of airway instability rather than an incidental bystander.

Sengupta *et al*. (2025) [7] further highlighted its clinical relevance in adults with pre-existing pulmonary disease, demonstrating recovery of pure growth *M. catarrhalis* from good-quality lower respiratory specimens in symptomatic patients who improved on directed therapy. Earlier syntheses by Verduin *et al.* (2002) [3] and Perez and Murphy (2017) [4] also emphasized its strong association with elderly pneumonia and COPD exacerbations. Against this background, the study was undertaken to determine the proportion of *M. catarrhalis* among bacterial isolates recovered from lower respiratory tract specimens, to analyze associated demographic and clinical risk factors, and to evaluate the antimicrobial susceptibility pattern and phenotypic clavulanate synergy screening results of the isolates.

## Materials and methods

This prospective observational laboratory-based study was conducted in the Department of Microbiology at Meenakshi Medical College Hospital and Research Institute, Kanchipuram, Tamil Nadu, India, over a 13-month period from December 2024 to December 2025. The study protocol was approved by the Institutional Ethics Committee of Meenakshi Medical College Hospital and Research Institute under approval number MMCH & RI IEC/PG/09/Nov/2024.

Study population and specimen selection

Respiratory specimens were collected from patients with clinically suspected lower respiratory tract infection. The study specimens included sputum, bronchoalveolar lavage, and bronchial washing samples submitted to the microbiology laboratory during the study period. Sputum specimens were screened for bacteriological acceptability using Bartlett’s grading system, and only samples with a Bartlett score [8] of ≥1 were included for culture processing, thereby strengthening specimen quality in the study. Bronchoalveolar lavage and bronchial washing specimens obtained for evaluation of lower respiratory tract infection were also included. Pediatric patients, patients unwilling to participate, and sputum specimens with a Bartlett score of <1 were excluded. A total of 100 eligible respiratory specimens were included in the final analysis.

Specimen collection processing and identification of *M. catarrhalis*

All specimens were collected and processed using standard aseptic microbiological procedures. After receipt in the laboratory, eligible samples were inoculated onto blood agar and incubated aerobically at 37°C for 16 to 18 hours. Following incubation, suspected colonies were examined for colony morphology and subjected to Gram staining and standard biochemical testing. Isolates suspected to be *Moraxella *species were identified phenotypically and verified by the hockey puck test. The final isolate classification used in the study was based on these conventional phenotypic laboratory methods.

Antimicrobial susceptibility testing

Antimicrobial susceptibility testing of *M. catarrhalis* isolates was performed using the Kirby-Bauer disk diffusion method following routine laboratory procedures and interpreted according to the laboratory’s adopted Clinical and Laboratory Standards Institute (CLSI)-based criteria. For each isolate, a bacterial suspension was prepared in sterile phosphate-buffered saline and adjusted to 0.5 McFarland turbidity. The standardized inoculum was lawn cultured onto Mueller-Hinton agar, after which antibiotic discs were applied. The antimicrobial agents tested were amoxicillin-clavulanate (20/10 µg), ampicillin (10 µg), azithromycin (15 µg), cotrimoxazole (1.25/23.75 µg), ciprofloxacin (5 µg), and cefotaxime (30 µg). Zone diameters were interpreted as susceptible, intermediate, or resistant according to CLSI criteria. In the finalized dataset, minimum inhibitory concentration-based susceptibility categories were also tabulated descriptively alongside disk diffusion results in the Results section.

Detection of extended-spectrum beta-lactamase production

Screening for extended-spectrum beta-lactamase production among *M. catarrhalis* isolates was performed using third-generation cephalosporins. Phenotypic confirmation was performed using the combination disk test with cefotaxime and ceftazidime, alone and in combination with clavulanic acid. An increase in inhibition zone diameter of ≥5 mm for the cephalosporin-clavulanic acid combination compared with the cephalosporin alone was interpreted as positive for ESBL production.

Outcome measures

The primary outcome measure was the proportion of *M. catarrhalis* among bacterial isolates recovered from lower respiratory tract specimens. Secondary outcome measures included the age and sex distribution of affected patients, associated clinical conditions, underlying risk factors, antimicrobial susceptibility profile, and frequency of ESBL production among the isolates.

Statistical analysis

Data were analyzed using IBM SPSS Statistics for Windows, Version 29 (Released 2023; IBM Corp., Armonk, New York). Categorical variables were expressed as frequency and percentage. Exploratory chi-square goodness-of-fit testing was used to evaluate the distribution of bacterial isolate categories, age groups, associated clinical conditions, and risk factors. Exact binomial testing was applied for sex distribution among *M. catarrhalis* isolates. A p-value of <0.05 was considered statistically significant. Because only aggregated susceptibility counts were available, formal isolate-level paired comparison between disk diffusion and minimum inhibitory concentration categories was not performed, and antimicrobial susceptibility findings were reported descriptively.

## Results

A total of 100 respiratory specimens were included in the study, yielding 100 bacterial isolates. Of these, 16 isolates (16.0%) were identified as *M. catarrhalis*. The remaining isolates comprised *Klebsiella pneumoniae* (n = 22, 22.0%), *Acinetobacter baumannii *(n = 20, 20.0%), *Streptococcus pneumoniae *(n = 16, 16.0%), *Staphylococcus aureus* (n = 14, 14.0%), *Haemophilus influenzae* (n = 8, 8.0%), and* Burkholderia species *(n = 4, 4.0%). An exploratory chi-square goodness-of-fit test showed heterogeneity in isolate distribution across organism categories (χ² = 17.04, p = 0.009). Microscopy demonstrated gram-negative diplococci, and colony morphology showed 1-2 mm, grayish-white, opaque, circular, convex, non-hemolytic colonies with a positive hockey puck sign (Table 1).

**Table 1:** Distribution of bacterial isolates recovered from lower respiratory tract specimens (N = 100) *p < 0.05 was considered statistically significant. Values are presented as n (%). The p-value was obtained using an exploratory chi-square goodness-of-fit test across isolate categories.

Bacterial isolate	n (%)	Test statistic	p-value
Moraxella catarrhalis	16 (16.0)	χ² = 17.04	0.009*
*Burkholderia* species	4 (4.0)		
Staphylococcus aureus	14 (14.0)		
Streptococcus pneumoniae	16 (16.0)		
Klebsiella pneumoniae	22 (22.0)		
Acinetobacter baumannii	20 (20.0)		
Haemophilus influenzae	8 (8.0)		
Overall distribution	100 (100.0)		

Among the 16 *M. catarrhalis* isolates, nine cases (56.3%) occurred in males and seven (43.8%) in females. On exact binomial testing, the sex distribution was not statistically significant (p = 0.804). The highest age-group frequency was observed in patients aged >60 years (n = 6, 37.5%), followed by 31-40 years (n = 4, 25.0%), 51-60 years (n = 3, 18.8%), 41-50 years (n = 2, 12.5%), and <30 years (n = 1, 6.3%); this age distribution was not statistically significant on exploratory testing (χ² = 4.63, p = 0.328). With respect to associated clinical conditions, COPD was the most common diagnosis (n = 5, 31.3%), followed by pneumonia (n = 4, 25.0%), pulmonary tuberculosis (n = 3, 18.8%), bronchiectasis (n = 2, 12.5%), empyema (n = 1, 6.3%), and lung carcinoma (n = 1, 6.3%) (χ² = 5.00, p = 0.416). Diabetes mellitus was the most frequent risk factor (n = 7, 43.8%), followed by smoking (n = 3, 18.8%), hypertension (n = 2, 12.5%), and alcoholism (n = 2, 12.5%), while two patients (12.5%) had no identifiable risk factor; this distribution was also not statistically significant on exploratory analysis (χ² = 5.88, p = 0.209) (Table 2).

**Table 2:** Demographic and clinical characteristics of patients with M. catarrhalis isolation (N = 16) Values are presented as n (%). Exact binomial testing was used for sex distribution. Exploratory chi-square goodness-of-fit testing was used for age-group, associated clinical condition, and risk-factor distributions. COPD: chronic obstructive pulmonary disease

Parameter	Category	n (%)	Test statistic	p-value
Sex	Male	9 (56.3)	Exact binomial test	0.804
	Female	7 (43.8)		
Age group	<30 years	1 (6.3)	χ² = 4.63	0.328
	31–40 years	4 (25.0)		
	41–50 years	2 (12.5)		
	51–60 years	3 (18.8)		
	>60 years	6 (37.5)		
Associated clinical condition	COPD	5 (31.3)	χ² = 5.00	0.416
	Pneumonia	4 (25.0)		
	Pulmonary tuberculosis	3 (18.8)		
	Bronchiectasis	2 (12.5)		
	Empyema	1 (6.3)		
	Lung carcinoma	1 (6.3)		
Risk factors	Diabetes mellitus	7 (43.8)	χ² = 5.88	0.209
	Smoking	3 (18.8)		
	Hypertension	2 (12.5)		
	Alcoholism	2 (12.5)		
	No identifiable risk factor	2 (12.5)		

Antimicrobial susceptibility testing showed the highest resistance to ampicillin, with seven isolates (43.8%) classified as resistant. Resistance to ciprofloxacin was observed in five isolates (31.3%), and resistance to cefotaxime in three isolates (18.8%). Resistance to cotrimoxazole and amoxicillin-clavulanate was observed in one isolate (6.3%) each. All 16 isolates (100.0%) were susceptible to azithromycin. These findings are presented descriptively (Table 3).

**Table 3:** Antimicrobial susceptibility pattern of M. catarrhalis isolates by Kirby-Bauer disk diffusion (N = 16) Values are presented as n (%). Susceptibility categories were determined by Kirby-Bauer disk diffusion and are shown descriptively.

Antibiotic	Disk diffusion resistant n (%)	Disk diffusion intermediate n (%)	Disk diffusion sensitive n (%)
Ampicillin (10 µg)	7 (43.8)	2 (12.5)	7 (43.8)
Ciprofloxacin (5 µg)	5 (31.3)	1 (6.3)	10 (62.5)
Cefotaxime (30 µg)	3 (18.8)	1 (6.3)	12 (75.0)
Cotrimoxazole (1.25/23.75 µg)	1 (6.3)	2 (12.5)	13 (81.3)
Amoxicillin-clavulanate (20/10 µg)	1 (6.3)	0 (0.0)	15 (93.8)
Azithromycin (15 µg)	0 (0.0)	0 (0.0)	16 (100.0)

Extended-spectrum beta-lactamase production was confirmed in one of the 16* M. catarrhalis* isolates, corresponding to an overall ESBL positivity rate of 6.3%. The same isolate was identified by confirmatory testing with both cefotaxime-clavulanic acid and ceftazidime-clavulanic acid combinations (Table 4).

**Table 4:** Detection of extended-spectrum beta-lactamase production in M. catarrhalis by combination disk test (N = 16) Values are presented as n (%). This table is descriptive. ESBL positivity was defined as an increase in inhibition zone diameter of ≥5 mm for the antimicrobial agent tested in combination with clavulanic acid compared with the cephalosporin alone. The overall ESBL positivity rate was 1/16 (6.3%).

Antibiotic disk	Confirmatory disk combination	Positive isolates n (%)
Cefotaxime	Cefotaxime + clavulanic acid (30/10 µg)	1 (6.3)
Ceftazidime	Ceftazidime + clavulanic acid (30/10 µg)	1 (6.3)

## Discussion

The present study reinforces the growing view that *M*. *catarrhalis* should no longer be regarded as a casual respiratory bystander in adult lower respiratory tract infections. In this cohort, *M*. *catarrhalis* accounted for 16.0% of all bacterial isolates, placing it among the notable recovered pathogens rather than at the microbiological margins. Descriptively, *M. catarrhalis* isolates were more frequently observed in patients older than 60 years and in males, while COPD was the most common associated clinical condition, and diabetes mellitus was the most frequent accompanying risk factor. Equally important, the antimicrobial profile was not benign: ampicillin resistance was highest at 43.8%, ciprofloxacin resistance reached 31.3%, cefotaxime resistance was 18.8%, and one isolate showed clavulanate synergy positivity suggestive of an expanded beta-lactam resistance phenotype. At the same time, all isolates remained susceptible to azithromycin, and most retained susceptibility to amoxicillin-clavulanate. These findings collectively portray *M*. *catarrhalis* as a clinically meaningful opportunistic pathogen in structurally compromised adult airways, albeit one whose therapeutic vulnerability remains partly preserved.

The broad direction of these findings aligns well with existing literature. Verduin* et al.* (2002) [3] and Perez and Murphy (2017) [4] framed *M. catarrhalis* as an established respiratory pathogen, particularly among older adults and those with chronic airway disease. The present observation that COPD was the most frequent associated clinical condition is consonant with studies by Aiswariya *et al.* (2017) [9] and Latheef *et al.* (2025) [10], all of which identified COPD as a dominant clinical context for isolation. Likewise, the age skew toward older patients mirrors that of Hirai *et al*. (2020) [11], who showed that *M. catarrhalis* community-acquired pneumonia has a distinctly elderly, comorbidity-rich profile, and Yagyu *et al.* (2025) [6], who linked prior airway infection with this organism to recurrent COPD hospitalization. Thus, the current data fit a consistent epidemiological pattern: *M. catarrhalis* tends to surface where airway defense is already weakened, whether by age, chronic inflammation, structural distortion, or metabolic vulnerability.

The antimicrobial findings show partial agreement, but also clinically revealing divergence, from prior reports. The observed ampicillin resistance supports the long-recognized beta-lactamase-associated resistance burden described by Verduin *et al.* (2002) [3], Gupta *et al.* (2011) [12], and Ramadan *et al. *(2017) [13]. However, the magnitude here was lower than the 71% to >90% resistance reported in several earlier series. Similarly, the preservation of azithromycin susceptibility contrasts sharply with pediatric and some regional studies, such as Du *et al.* (2017) [14], Xiang *et al.* (2025) [15], and Zhao *et al.* (2022) [16], where macrolide non-susceptibility was far more prominent. These disagreements are biologically plausible rather than contradictory. First, resistance ecology in *M. catarrhalis* is highly geography-dependent. Second, adult lower respiratory isolates may not mirror pediatric carriage or pediatric pneumonia populations, where antibiotic exposure and clonal circulation differ. Third, the small isolate count in the present study magnifies local prescribing effects and sampling variation. Finally, the absence of molecular characterization means that BRO beta-lactamase subtypes, macrolide resistance determinants, and clonal background remain unknown. In other words, the study captures a real local phenotype, but not the full genomic architecture underneath it.

A further point deserving emphasis is that the numerical predominance of older age, COPD, and diabetes did not achieve statistical significance. This should not be ignored or cosmetically softened. Rather, it likely reflects limited power from the small number of *M. catarrhalis* isolates. The pattern is clinically persuasive and literature-concordant, but inferential restraint remains essential. That distinction matters because it prevents the discussion from slipping into overstatement while still allowing biologically coherent interpretation.

Clinically, the implications are straightforward. Although *M. catarrhalis* may colonize chronically diseased airways, particularly in patients with underlying airway pathology, its recovery from good-quality lower respiratory specimens in symptomatic adults should be interpreted in the clinical context rather than being dismissed automatically as colonization. In the present setting, this was especially relevant in patients with COPD, pneumonia, bronchiectasis, prior tuberculosis, or diabetes. Routine susceptibility testing appears justified, particularly because empirical ampicillin would be unreliable in this setting, and ciprofloxacin resistance was not trivial. The low resistance to amoxicillin-clavulanate and preserved azithromycin susceptibility are reassuring, but stewardship still demands local antibiogram-based therapy rather than textbook assumptions. The clavulanate synergy-positive isolate also argues for continued surveillance of evolving beta-lactam resistance phenotypes.

This study has several limitations: single-center design, modest sample size, lack of molecular confirmation or BRO gene analysis, dependence on conventional phenotypic identification, absence of detailed seasonal and outcome correlations, and a descriptive rather than isolate-level comparative analysis for disk diffusion versus MIC categories. The ESBL-like finding also requires cautious interpretation without genotypic validation.

## Conclusions

The present study strengthens the evidence that *M. catarrhalis* is a clinically relevant lower respiratory tract pathogen in adults, particularly among elderly patients and those with underlying comorbid airway disease. Its observed resistance to ampicillin, alongside preserved susceptibility to azithromycin and largely retained activity of amoxicillin-clavulanate, highlights the need for careful microbiological identification and locally guided antimicrobial selection. Although the resistance profile was not uniformly severe, the observation of phenotypic clavulanate synergy positivity in one isolate supports continued surveillance for evolving beta-lactam resistance patterns. Taken together, these findings underscore that *M. catarrhalis* should be regarded not as a trivial colonizer but as a meaningful respiratory pathogen with tangible diagnostic and therapeutic implications.
